# Functional Overexpression of Vomeronasal Receptors Using a Herpes Simplex Virus Type 1 (HSV-1)-Derived Amplicon

**DOI:** 10.1371/journal.pone.0156092

**Published:** 2016-05-19

**Authors:** Benjamin Stein, María Teresa Alonso, Frank Zufall, Trese Leinders-Zufall, Pablo Chamero

**Affiliations:** 1 Department of Physiology, and Center for Integrative Physiology and Molecular Medicine, University of Saarland School of Medicine, Homburg, Germany; 2 Instituto de Biología y Genética Molecular (IBGM), University of Valladolid and CSIC, Valladolid, Spain; 3 Laboratoire de Physiologie de la Reproduction & des Comportements, UMR85 INRA-CNRS-IFCE-Université de Tours, Nouzilly, France; Monell Chemical Senses Center, UNITED STATES

## Abstract

In mice, social behaviors such as mating and aggression are mediated by pheromones and related chemosignals. The vomeronasal organ (VNO) detects olfactory information from other individuals by sensory neurons tuned to respond to specific chemical cues. Receptors expressed by vomeronasal neurons are implicated in selective detection of these cues. Nearly 400 receptor genes have been identified in the mouse VNO, but the tuning properties of individual receptors remain poorly understood, in part due to the lack of a robust heterologous expression system. Here we develop a herpes virus-based amplicon delivery system to overexpress three types of vomeronasal receptor genes and to characterize cell responses to their proposed ligands. Through Ca^2+^ imaging in native VNO cells we show that virus-induced overexpression of *V1rj2*, *V2r1b* or *Fpr3* caused a pronounced increase of responsivity to sulfated steroids, MHC-binding peptide or the synthetic hexapeptide W-peptide, respectively. Other related ligands were not recognized by infected individual neurons, indicating a high degree of selectivity by the overexpressed receptor. Removal of G-protein signaling eliminates Ca^2+^ responses, indicating that the endogenous second messenger system is essential for observing receptor activation. Our results provide a novel expression system for vomeronasal receptors that should be useful for understanding the molecular logic of VNO ligand detection. Functional expression of vomeronasal receptors and their deorphanization provides an essential requirement for deciphering the neural mechanisms controlling behavior.

## Introduction

In mice and other terrestrial vertebrates, social behavior and reproductive physiology are mediated by chemosignals that are recognized by the olfactory systems [1,2]. The accessory (or vomeronasal) olfactory system seems to specialize in social interactions such as those conveyed by pheromones, kairomones, and other socially relevant chemosignals. These molecular cues are detected in the vomeronasal organ (VNO) by sensory neurons that express three major families of G protein-coupled receptors (GPCRs), vomeronasal receptors (*VRs*) type 1 and 2 (*V1Rs* and *V2Rs*), and formyl peptide receptors (*Fprs*) [3–6]. These receptors have been implicated in recognizing a highly diverse collection of ligands and initiating transduction in vomeronasal sensory neurons (VSNs). However, only few receptors from a repertoire of nearly 400 functional genes have been paired with molecularly defined ligands. In part, this is a direct consequence of the lack of a suitable expression system to enable in vitro receptor analysis of ligand specificity. Gene transfer and functional expression of cloned vomeronasal receptors is a fundamental requirement for providing a causal link between receptor activation and ligand recognition and understanding the chemical receptive properties that, in turn, provide the foundation for neural coding of social odorants.

Identification of a few vomeronasal receptor-ligand pairings has been possible by using gene-targeted mice that express fluorescent markers in specific sensory neurons and, in some cases, through genetic deletion of receptors. Known receptor-ligand pairs include *V1rj2* and *V1rj3* paired with sulfated steroids [7], *V1rb2* paired with 2-heptanone [8], *V2rp5* paired with ESP1 peptide [9], and *V2r1b* and *V2rf2* paired with 9-mer peptides [10,11]. Despite recent advances in genome editing, generation of knockin and knockout mice is still costly and time consuming, and in consequence impractical to cover the enormous vomeronasal receptor repertoire. As a rare exception, two *V2Rs* (*V2rp1* and *V2rp2*, which also detect ESP peptides) and one *Fpr* (*Fpr3*, previously known as *Fpr-rs1*, detecting bacterial signal peptides) have been functionally expressed in heterologous cells [12–15]. To our knowledge, however, this approach was not effectively used with other *VRs* and *Fprs*, thus far. A rapid and uncomplicated expression system that covers any vomeronasal receptor type would provide an invaluable tool for understanding the logic of information processing in the VNO.

A functional expression system for vomeronasal receptors requires first that the receptors are properly localized to the surface of the cells, and second that they couple efficiently with a second messenger system that generate a measurable response to ligand stimulation. We reasoned that native VSNs themselves would be the most competent cells for expressing, localizing, and coupling vomeronasal receptors. Further support for this assumptions comes from the existence of only two major signaling cascades in the VNO: one that involves the G-protein Gαi2 used by *V1R*-expressing VSNs that are localized in the apical VNO layer, and a second one that involves Gαo in basal, V2R-positive VSNs [16–19]. Fpr signaling also seems to follow similar segregation rules as determined by G-protein expression: Fpr-rs3, 4, 6 and 7 co-localize with Gαi2 whereas Fpr3 co-localizes with Gαo [20]. Here, we rely on the large number of native VSNs expressing either Gαi2 or Gαo to develop an expression system, driving the expression of selected members from each of the *V1R*, *V2R* or *Fpr* families by delivering them through a herpes virus amplicon vector and infecting VSNs in vitro. Infected individual cells are identified by bicistronic expression of marker green fluorescent protein (GFP), allowing responses to specific ligands to be tested in single cells. Ca^2+^ imaging recordings show that ectopic expression of a single *V1R*, *V2R* or *Fpr* gene causes a significant increase in the number of responses to cognate ligands in VSNs overexpressing one of these receptors. Our results provide a simple and effective system for functional expression of three types of vomeronasal receptors that should be useful in the deorphanization effort of these important receptor families.

## Methods

### Mice

Animal care and experimental procedures were performed in accordance with the German Animal Welfare Act, European Communities Council Directive 2010/63/EU, and the institutional ethical and animal welfare guidelines of the University of Saarland (approval number of the Institutional Animal Care and Use Committee: H-2.2.4.1.1). Male C57Bl/6, OMP-GFP mice (B6.*OMP*^*tm3Mom*^/MomJ; originally JR# 006667) [21] and Gnao1/OMP-Cre conditional Gαo knockout [22–24] mice were used. Mice were 6–15 weeks old and were kept under standard light/dark cycle (12:12) with food and water *ad libitum*. Mice were euthanized by CO_2_ asphyxiation followed by cervical dislocation as directed by the guidelines of the University of Saarland.

### VSN primary culture

**Fig 1:**
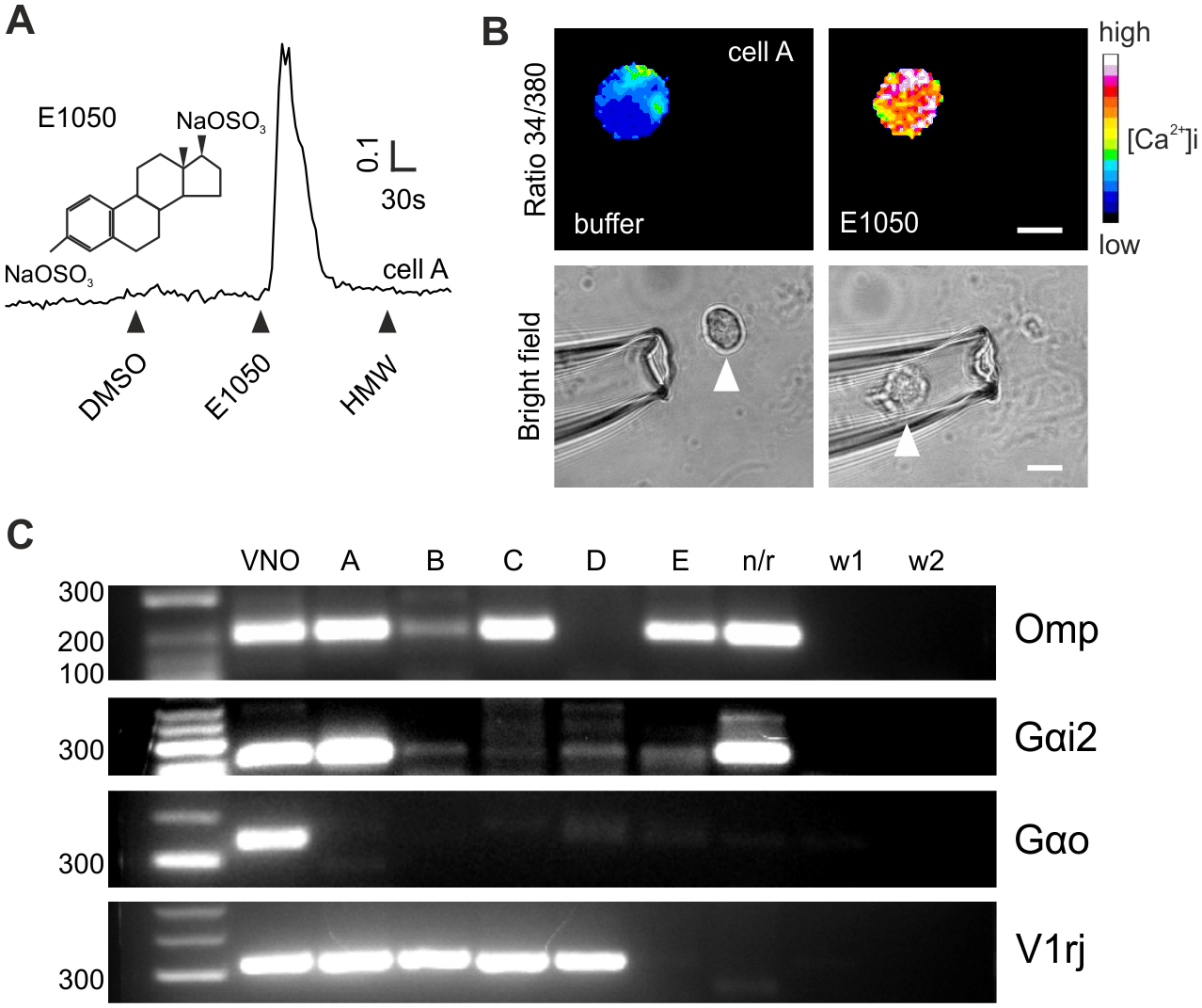
Single-cell RT-PCR following Ca^2+^ imaging and cell isolation. (**A**) Representative intracellular Ca^2+^ increase (F340/380 ratio, arbitrary units) of a VSN (cell “A”) loaded with fura-2 in response to the sulfated steroid E1050 (chemical structure shown on the side), but not to urine high molecular weight fraction (HMW). (B) Ratiometric (340/380) imaging of the cell shown in A during stimulation with control buffer (DMSO) and E1050. Responsive cells are later picked using a glass capillary micropipette. Arrowhead points to the cell before and after (inside of the micropipette) picking. Scale bar, 10 μm. (C) Ethidium-bromide stained agarose gels of RT-PCR products generated from 5 cells (A to E) showing Ca^2+^ responses to E1050, a single cell lacking responses (n/r), and two water controls (w1 and w2). cDNA collected from pooled whole VNOs was used as positive control (VNO). PCR amplification of cDNA collected from single cells was performed using gene specific primers for *Omp*, *Gnao1* (Gαo), *Gnai2* (Gαi2) and degenerate primers for three members of the *V1rj* family.

To obtain freshly dissociated VSNs, the mouse VNO epithelium was detached from the cartilage and minced in PBS at 4°C. The tissue was incubated (20 min at 37°C) in PBS supplemented with papain (0.22 U/ml) and DNase I (10 U/ml; Fermentas), gently washed and centrifuged (100 × g, 5 min) in Dulbecco’s modified Eagle’s medium (DMEM, Invitrogen) supplemented with 10% fetal bovine serum (FBS; Gibco). Dissociated cells were plated for 1 h on coverslips previously coated with concanavalin-A type V (0.5 mg/ml, overnight at 4°C; Sigma). Cells were either used immediately for imaging (Fig 1), or infected with virus and incubated at 37°C in FBS-supplemented DMEM medium for 20–24h before imaging.

### Calcium imaging

Imaging was performed as described [22,24,25]. Briefly, cells were loaded with the Ca^2+^ indicator fura-2/AM (5 μM; Invitrogen). Coverslips containing fura-2-loaded VSNs were placed in a laminar-flow chamber (Warner Instruments) and constantly perfused with extracellular Hank’s balanced salt solution (HBSS) supplemented with 10mM Hepes (2-[4-(2-hydroxyethyl)piperazin-1-yl]ethanesulfonic acid). Calcium imaging on HSV-infected cells was performed 20–24 h post infection. Ratiometric (F340/F380) fura-2 Ca^2+^ imaging was performed using an Olympus IX71 microscope equipped with a Hamamatsu camera. Image pairs were acquired at 0.25 Hz and analyzed using ImageJ (NIH). Chemostimuli were bath applied for 30 s. Image processing and data analysis, including region of interest (ROI) detection and signal analyses, were performed using ImageJ (NIH) and Origin8.6 (OriginLab). ROIs were selected manually and peak signals calculated from the temporal profiles of image ratio values. A response was defined as a stimulus-dependent deviation in fluorescence ratio that exceeds 1.5 the standard deviation of the mean of the baseline.

### Chemostimulation

**Fig 2:**
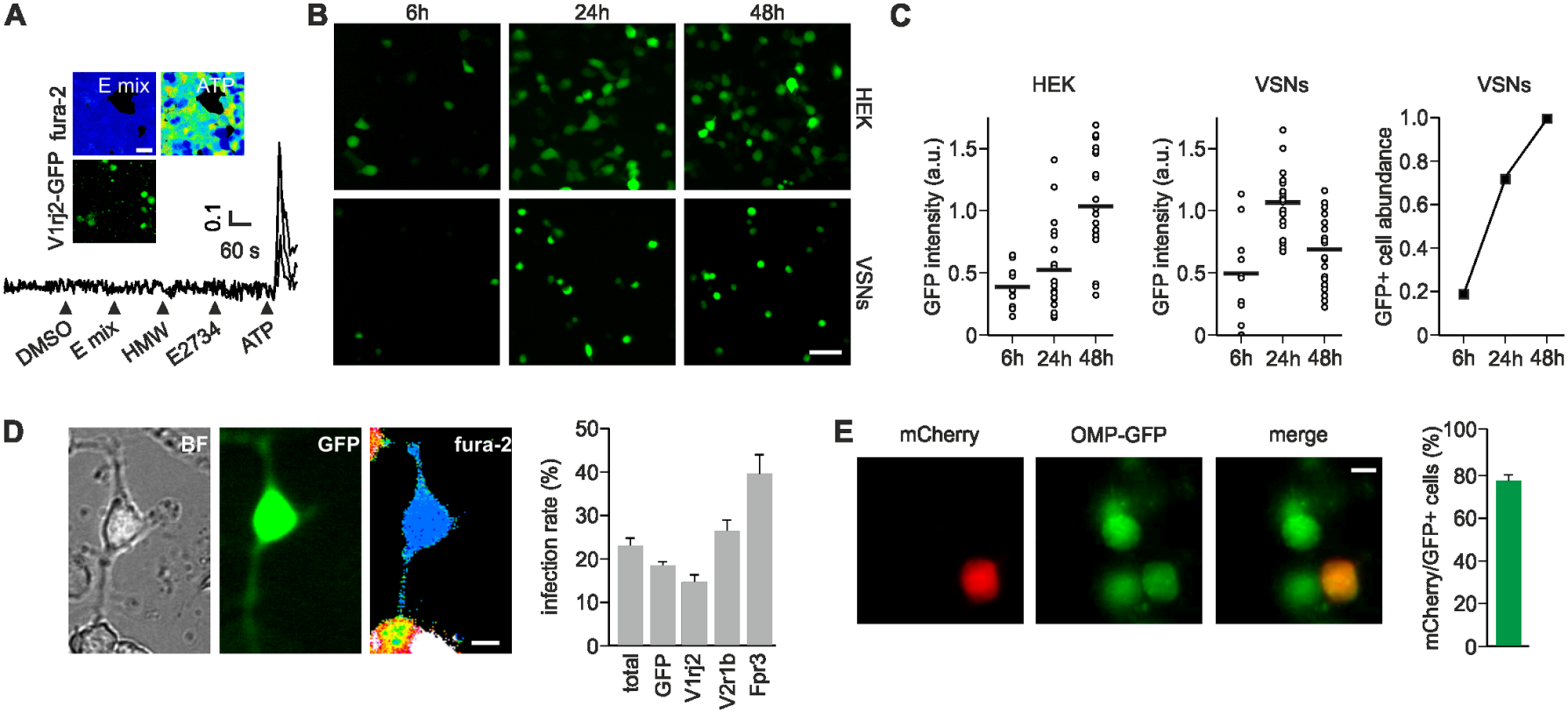
Herpes simplex virus type 1 (HSV-1) expression system in HEK cells and VSNs. (A) HEK cells transfected with pHSV-*V1rj2*-IRES-*GFP* expression vector do not show responses to a mix of sulfated steroids (E mix: E1100, E0893, E0588, and E1050, each 100 μM), HMW nor E2734 in Ca^2+^ imaging. (B) Infection of HEK cells (top panels) and freshly prepared VSNs (bottom panels) with HSV-GFP amplicon virus monitored at three different time points (6 h, 24 h and 48 h). Scale bars, 50 μm (C) Left and center, measures of fluorescence intensity (in arbitrary units, a.u.) on infected single HEK cells and VSNs. Right, normalized abundance of infected VSNs (GFP+) at each time point. (D) Single VSNs infected with HSV-GFP virus for 20 h and loaded with fura-2. Bright field (BF), GFP and F340/380 ratio images of an infected cell are shown. Average rate of infection was 23% (N = 16 402 cells in 69 infections). Infection rate for specific batches of HSV: GFP, 19% (N = 10 171); V1rj2, 15% (N = 3109); V2r1b, 27% (N = 2675); Fpr3, 39% (N = 447). (E) VSNs prepared from OMP-GFP mice infected with a HSV-mCherry virus. Of all mCherry-positive cells 76% were also positive for GFP. N = 978 mCherry+ cells in 14 infections. Scale bars, 10 μm.

**Fig 3:**
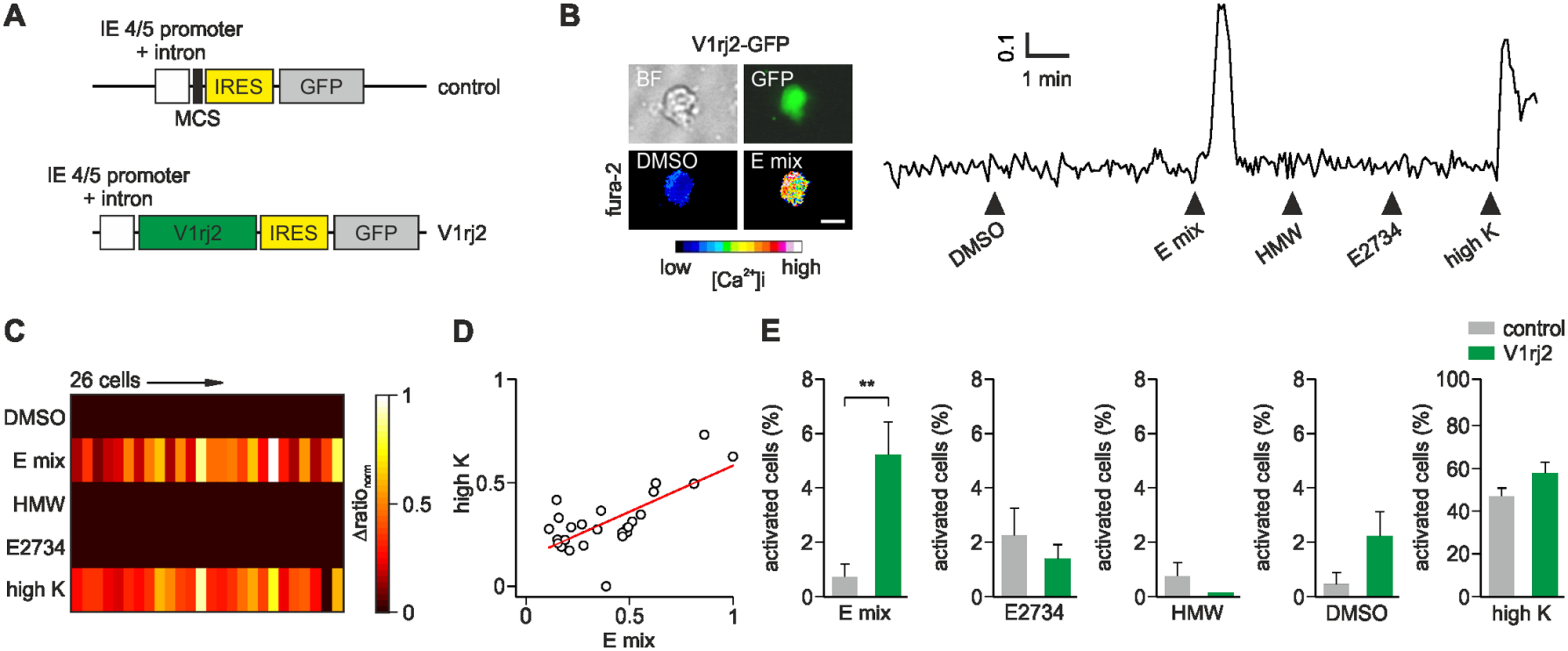
Functional overexpression of *V1rj2* in HSV-infected VSNs. (A) Diagram of the HSV-1 expression cassettes. In control amplicons *GFP* is expressed under the HSV-1 IE4/5 promoter, whereas in V1rj2 amplicons *V1rj2* cDNA is inserted in the multi cloning site (MCS) upstream of IRES and GFP. (B) Bright field (BF), GFP and pseudocolor fura-2 images of a representative VSN infected with HSV-*V1rj2*-IRES-*GFP* and activated by the mix of four sulfated estrogens (E mix). Time course of intracellular calcium is shown on the right. Stimulations were 30 s long. Scale bar, 10 μm. (C) Color-coded heat map of normalized Δratio responses (Δratio_norm_) from 26 GFP+ VSNs infected with *V1rj2*-IRES-*GFP* which showed responses to the estrogen mix (E mix). These cells lacked responses to other stimuli except for high K^+^. (D) Comparison of response amplitudes expressed as normalized Δratio responses to E mix and high K of 26 individual cells shown in C. Each neuron is shown as a separate circle. Red line represents linear fit (slope = 0.55). Responses to E mix and high K^+^ are not significantly different (p = 0.92, Mann-Whitney test). (E) Summary of GFP+ cell activation for every stimulus on VSNs infected either with V1rj2-GFP (green) or with GFP control amplicons (grey). Values are expressed as percentage (%) of activated cells from the total of GFP+ cells. VSNs expressing *V1rj2-GFP* show increased cell responsivity to E mix (p < 0.005) but not to E2734, HMW, DMSO or high K^+^ (p = 0.1–0.4, Student’s t test). Responses to DMSO, E2734 or HMW did not overlap with responses to E-mix. N = 436 *V1rj2-GFP* cells and 563 control-*GFP* cells, in 8 and 16 experiments, respectively.

Chemostimuli for Ca^2+^ imaging were prepared fresh daily and diluted in Hepes-buffered HBSS, giving the following final concentrations: SYFPEITHI, 5×10^−11^ M; urine high molecular fraction (HMW) 1:300 dilution; sulfated estrogen mix (E mix): E1050 (1, 3, 5(10)-estratrien-3, 17β-diol disulphate), E1100 (1, 3, 5(10)-estratrien-3, 17β-diol 3-sulphate), E0893 (1, 3, 5(10)-estratrien-3, 17α-diol 3-sulphate) and E0588 (17β-dihydroequilin D 3-sodium sulphate), each at 100 μM (Steraloid); E2734 (1, 3, 5(10)-estratrien-3, 16α, 17β-triol 17-sulphate), 100 μM; W-peptide (WKYMVm), 1μM; mitochondria-derived ND1-peptide (f-MFFINTLTL), 10^−7^ M; KCl, 100 mM; ATP (adenosine triphosphate), 60 μM. Sulfated estrogens were prepared in DMSO (dimethyl sulfoxide) and further diluted into Hepes-HBSS. A solution containing the same amount of DMSO (labeled as DMSO in Figs 1–3) was used as a control stimulation for E1050 or the sulfated estrogen mix. To obtain HMW fraction, fresh urine was collected from adult C57Bl/6 males (8–12 week old, sexually naïve) and size-fractionated by centrifugation (14,000 × g for 30 min) using Microcon 10-kDa molecular mass cutoff ultrafiltration columns (Millipore). The centrifugation retentate was washed with one volume of PBS three times and resuspended in PBS to the initial volume of urine. The MHC-peptide SYFPEITHI was initially dissolved in a solution containing: 120 mM NaCl, 25 mM NaHCO_3_, 5 mM KCl, 5 mM N,N-bis[2-hydroxyethyl]-2-aminoethanesulfonic acid (BES), 1 mM MgSO_4_, 1 mM CaCl_2_, 10 mM glucose] to give 100 μM stock solutions. Further dilutions were made in Hepes-buffered HBSS.

### Single-cell RT-PCR

Single-cell cDNAs were reverse transcribed and amplified by a previously described procedure [26]. VSNs exhibiting a specific response profile identified in Ca^2+^ imaging were picked using a glass capillary (~10 μm tip size) in 1 μl of Hepes-HBSS. Single cells were then transferred to a PCR tube containing 1μl of diethylpyrocarbonate (DEPC) treated water. Samples were immediately frozen on dry ice and kept at −80°C until use. Cells were lysed in 5μl of a NP-40 based lysis buffer by incubation at 65°C for 1min. Reverse transcription (RT) was performed with anchor-T primer (TATAGAATTCGCGGCCGCTCGCGA[T]24) and Superscript III (Invitrogen) at 37°C for 90 min and at 50°C for 15 min. Reactions were stopped by heating at 70°C for 10 min. Next, a tailing reaction was performed using TdT, dATP and RNAseH (Roche) at 37°C for 20 min, followed by incubation at 65°C for 10 min. For analysis of gene expression, RT-tailed products were first amplified by PCR reaction with EX-Taq HS DNA polymerase (Takara) and anchor-T primer by incubating at 95°C for 2 min, 37°C for 5 min, 72°C for 20 min, then 30 cycles of 95°C for 30 s, 67°C for 1 min, 72°C for 6 min plus 6 s extension for each cycle, then 72°C for 10 min. The second round of PCR was with the following primers: V1rj: CATCACTCCCAGTAAYTCTAAGTGGGC and GCTGGGMTCTCTTGTRRTGYCTGTAGAG; *Gnai2*: GAGCATGAAGCTGTTTGACAGC and CTCCTTGGTGTCTTTGCGC; *Omp*: GCACAGTTAGCAGGTTCAGCT and GGTTTGCAGTCCTGGCAGC; *Gnao*: CTCCACGAGGACGAAACCAC and GCCCCGGAGATTGTTGGCA. V1rj primers are designed to capture *V1rj2* (*Vmn1r89*), *V1rj3* (*Vmn1r85*), and *Vmn1r86* genes. Total VNO mRNA was obtained from pooled C57Bl/6 adult mice (both genders) VNO tissue using PureLink RNA Mini Kit (Ambion) according to the manufacturer’s instructions. Traces of genomic DNA were digested by incubation with 30U of DNase I (Fermentas).

### Receptor cloning

*V1rj2 (Vmn1r89*), *V2r1b (Vmn2r24)* and *Fpr3* (previously known as *Fpr-rs1)* receptor cDNA cloning was performed by RT-PCR using C57Bl/6 mice pooled VNO mRNA and gene-specific primers: *V1rj2*: ATGTTTTCAAGTGACACATTCTTCCAGA and TCAGCCATGCAGTGAACTTTGATG; *V2r1b*: ATGAAATTACTCACTGCTTTCTCTCC and TCATTTAAAGAACTTCTTTCTTGAATG; *Fpr3*: ATGGAAACCAACTACTCTATCCCTTTGAAT and TATTGCCTTTATTTCAATGTCTTCAGGAAGT. PCR products were cloned first into TOPO or pGEM-T vectors and subcloned into pHSV-IRES-GFP vector followed by sequence analysis. The bicistronic pHSV-IRES-GFP amplicon was created by ligating the IRES-EGFP1 cassette previously excised with NotI/XbaI from the parental pIRES-GFP plasmid into the pHSVpUC amplicon linearized with the same restriction enzymes. pHSV-IRES-mCherry was generated by replacing EGFP1 sequence in the pHSV-IRES-GFP by mCherry cloned from pcDNA3.1/hChR2(H134R)-mCherry, a gift from Karl Deisseroth (Addgene plasmid # 20938).

### Cell lines and viruses

HEK293T (ATCC # CRL-11268) and 2–2 cells [27] were cultured in DMEM with 4.5 g/L D-glucose, supplemented with 10% FBS, 100 μg/ml penicillin and 10 μg/ml streptomycin. HEK cells were transfected with the mammalian expression vector pHSV-IRES-GFP containing *V1rj2* cDNA using lipofectamine LTX (Thermo Fisher Scientific) according to the manufacturer’s instructions. Herpes simplex virus type-1 (HSV-1) viral particles were prepared as described [28–30]. Briefly, pHSV-IRES-GFP and pHSV-IRES-mCherry empty vectors or containing receptor cDNAs were packaged into replication-deficient HSV-1 particles as follows: 2–2 cells were seeded on 60 mm dishes and transfected with the recombinant amplicon vectors using lipofectamine LTX (Thermo Fisher Scientific) for 24h. Then, medium was replaced and cells were infected with 5*dl*1.2 helper virus particles containing a partial deletion in the IE2 (*ICP27*) gene of HSV-1. On the following day, virus was harvested and subsequently amplified three times on fresh 2–2 cells. VSNs and HEK cells were infected with 1–25 μl of HSV-1 (2 x 10^5^–8 x 10^5^ viral particles ml^-1^).

### Statistics

Independent Student’s t-test was used for measuring the significance of difference between two independent distributions. Mann-Whitney test was used for comparative analysis of cell-to-cell response variability. Multiple groups were compared using either one-way or two-way analysis of variance (ANOVA) with Tukey’s test as a *post hoc* comparison. Analysis was performed using Origin8.6 (OriginLab) software. Unless otherwise stated, results are presented as means ± SEM.

## Results

### Receptor identification by Ca^2+^ imaging and single-cell RT-PCR

Chemosignals present in urine and other secretions are detected by VSNs using a signal cascade that ultimately activates the ion channel Trpc2, causing robust intracellular Ca^2+^ transients [3]. We used Ca^2+^ imaging to identify the VRs responsible for detecting specific chemosignals. We first targeted VSNs that showed robust Ca^2+^ responses to 1, 3, 5(10)-estratrien-3, 17β-diol disulfate (E1050) (Fig 1A and 1B), a urinary sulfated steroid previously shown to activate VSNs [7,31]. We used a dissociated VSN preparation [22,24,25] to have access to activated single neurons and capture the whole cell using a micro-capillary pipette (Fig 1B). Single cells were then lysed and used as substrate for reverse transcription (RT) and cDNA amplification, followed by PCR using primers for specific genes. Pooled VNO mRNA as well as randomly picked single cells were used as controls. We isolated five cells responding to E1050 (cells A to E), four of which yielded a PCR product that amplified *Omp* (Fig 1C), encoding an abundant cytosolic protein expressed in all mature VSNs [32]. In all cells, we also identified expression of *Gnai2*, which codes for G-protein subunit Gαi2 (Fig 1C), typically expressed together with *V1R* receptors in the apical layer. VSNs responding to E1050 have been previously shown to express *V1Rs* of the *V1rj* subfamily [7,31]. Thus, we used a subset of degenerate primers designed to capture genes of this family: *V1rj2* (*Vmn1r89*), *V1rj3* (*Vmn1r85*), and *Vmn1r86*. We identified *V1rj* transcripts (either *V1rj2* or *V1rj3*) in four of the E1050-activated cells, but not in randomly picked cells (n/r) which were not activated by E1050 (Fig 1C). Cells were further screened for *Gnao1* encoding Gαo—which is not present in *V1R*-expressing VSNs—and found no detectable expression. Thus, using a combination of Ca^2+^ imaging and single cell RT-PCR we were able to identify specific vomeronasal receptors expressed in a given cell.

### HSV-1 expression system in intact VSNs

Identifying cognate ligands for olfactory or vomeronasal receptors in vitro has been a major challenge [33], mostly because of the lack of a robust heterologous expression system. Among the different vomeronasal receptors, only two *V2R*s (*V2rp1* and *V2rp2*) and one Fpr (*Fpr3*, also known as *Fpr-rs1*) have been functionally expressed in HEK cells [12–14]. To determine whether heterologous expression of *V1R*s is feasible, we cloned *V1rj2* into a mammalian expression vector and transfected HEK cells. Further Ca^2+^ imaging analysis revealed that the transfected cells—labeled with a GFP reporter—failed to show any response to a mix of sulfated steroids (E1050, E1100, E0893, E0588, and E1050, each at 100 μM) (Fig 2A), indicating that functional *V1R* expression was not effective in these cells. To circumvent this problem, we aimed at performing ectopic expression of vomeronasal receptors in their native cellular environment, i.e. in intact VSNs. This method has proven effective in the past by using transgenic mice [7]. However, production of genetically altered mice is laborious and time consuming. We reasoned that the use of a viral vector system in vitro could significantly simplify *VR* overexpression in native VSNs.

To test this possibility, we attempted to maintain acutely dissociated VSNs in vitro for a period of time sufficient to enable receptor expression, transport and localization to the plasma membrane. We were able to keep primary VSNs in culture for 24–48 h with almost intact cell viability using standard cell culture conditions (see Methods). Longer incubation periods resulted in increased cell death. Therefore, an efficient viral vector should enable fast and robust expression under these conditions. We then examined expression at different time points in HEK cells infected with a herpes simplex virus type 1 (HSV-1) amplicon vector. Amplicons are defective, helper-dependent, highly versatile gene transfer vectors in which, in contrast to other widely used viral vectors, the amplicon genome does not carry protein-encoding viral sequences. Consequently, they are safe for the host and non-toxic for the infected cells, yet with the ability to transduce cells at exceedingly high efficiencies. Furthermore, the complete absence of virus genes provides space to accommodate very large foreign DNA sequences, including complete genes [34]. HSV-1 is also specifically effective for infection of VSNs because, under natural conditions, it targets the mouse VNO as a route for neuroinvasion of the rodent central nervous system [35]. Thus, HSV-1 derived amplicons are adequate candidate vectors for fast and robust ectopic receptor expression in VSNs.

In HEK cells infected with a HSV-1 carrying GFP, we observed GFP fluorescence after 6 h (Fig 2B), with a peak in fluorescence intensity after 48 h (Fig 2C). Next, we infected acutely dissociated VSNs with the HSV-GFP virus and analyzed GFP fluorescence after 6, 24 and 48 h. In VSNs, we also observed GFP expression after 6 h following infection with little or no cytotoxic effects (Fig 2B). The relative number of cells expressing GFP increased sharply at 24 h (from 19% to 72%) and then stabilized between 24 and 48 h (Fig 2C). Maximum GFP fluorescence intensity values were observed after 24 h and decreased at 48 h (Fig 2C), perhaps as a result of reduced cell fitness. Therefore, in subsequent VSN experiments we used a 20–24 h interval for optimal infection efficiency.

We evaluated the integrity of the plasma membrane by loading the cells with the Ca^2+^ indicator fura-2 and observed robust dye uptake after 30 min of incubation (Fig 2D). On average, we observed an infection rate of 23% of all dissociated VNO cells in culture, measured as the number of cells positive for GFP (Fig 2D). VNO dissociation yields a mixed culture that includes VSNs but also glial supporting cells and immature VSNs. To determine whether VSNs were indeed infected, we used dissociated VNOs taken from OMP-GFP mice [21], which express GFP in all mature VSNs. GFP was detectable in ~ 80% of the cells in culture, thus unambiguously categorizing them as VSNs. Cells were then infected by HSV-1 carrying an mCherry expression cassette (Fig 2E). Among mCherry-positive cells, 76% were also positive for GFP (Fig 2E). Hence, HSV-1 was capable of infecting both VSNs and non-VSN cells with no evident bias for either cell type.

### Functional validation of V1R viral overexpression

Next, we asked whether *V1R*s overexpressed in virus-infected VSNs are functionally responsive. To address this question, we cloned *V1rj2* into an expression cassette of the HSV-1 amplicon (Fig 3A). To enable identification of infected cells, we included GFP in the expression cassette, inserting an internal ribosomal entry site (IRES) to produce a bicistronic transcript that results in the expression of *V1R* receptor and GFP as separate proteins in the same cells (Fig 3A). Ca^2+^ imaging assays on cells infected with the *V1rj2*-IRES-*GFP* virus revealed a 7-fold increase in the number of responsive cells (p < 0.005) to a mix of sulfated steroids (E1100, E0893, E0588, and E1050, each at 100 μM) as compared to responsive cells infected with the vector expressing *GFP* but no *V1rj2* (Fig 3B and 3E). Importantly, cells expressing *V1rj2* did not show significant Ca^2+^ responses to the related sulfated steroid E2734, major urinary proteins contained in the high molecular weight fraction of urine (HMW), or control buffer containing DMSO (p = 0.1–0.4) (Fig 3B, 3C and 3E). E2734 has been reported to show no activation of *V1rj2*-positive cells [31], and HMW was shown to activate V2R-positive VSNs [22]. Thus, the response profile of VSNs infected with *V1rj2*-encoding virus, at least within the scope of the ligands screened here, was relatively specific. We used a high (100 mM) K^+^ solution as positive control to estimate the number of infected VSNs and their viability. Receptor-GFP and GFP-only infected cells showed similar responses to high K^+^ solution (p = 0.12) indicating that ectopic expression of these receptors did not affect cell excitability and survival (Fig 3E). Both high K^+^ and E mix evoked similar calcium transient amplitudes in individual *V1rj2*-virus infected VSNs (Fig 3C and 3D), indicating that cell-to-cell response variability to E mix was comparable to that observed for high K^+^.

### V2Rs and Fprs functionally expressed in infected VSNs

**Fig 4:**
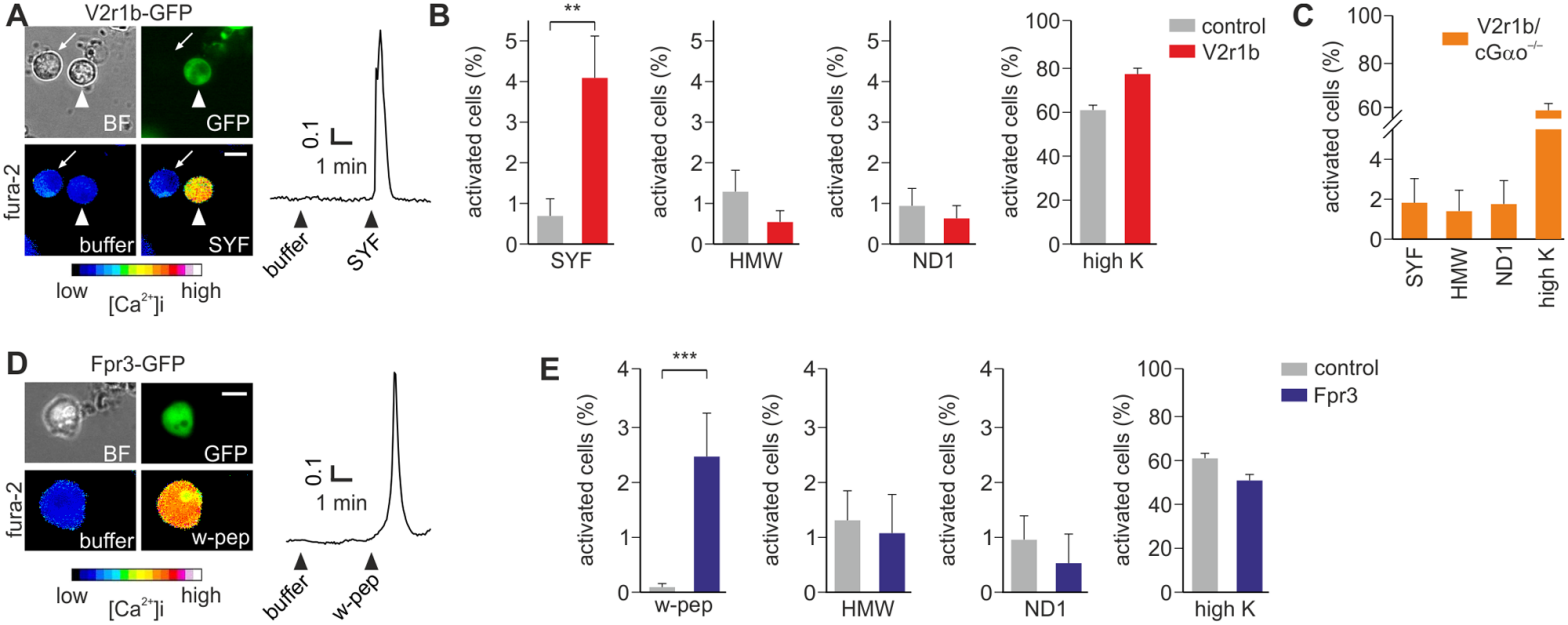
Viral transduction of *V2r1b* and *Fpr3* receptors in VSNs. (A) Bright field (BF), GFP and pseudocolor fura-2 images of a VSN infected with HSV-*V2r1b*-IRES-*GFP* (arrowhead) and activated by the MHC binding peptide SYFPEITHI (SYF). The neighboring non-infected GFP-negative cell (arrow) does not show any calcium increase during peptide stimulation. (B) Summary of cell responses to different stimuli. A 6-fold increase of responsivity to SYF is observed in *V2r1b-GFP* cells (p < 0.005, Student t test), but not to HMW fraction, the mitochondria-derived peptide ND1, or high K^+^. (C) Enhanced responsivity to SYF is not observed in Gαo-deficient mice (cGαo^-/-^) VSNs infected with HSV-*V2r1b*-IRES-*GFP*. (D) A single HSV-*Fpr3*-IRES-*GFP* infected VSN activated by the synthetic hexapeptide W-peptide (w-pep) is shown. (E) *Fpr3-GFP* cells show a significantly enhanced number of responses to W-peptide versus GFP control cells (p < 0.001, Student t test), but not to HMW, ND1 or high K^+^. N = 469 *V2r1b-GFP*, 163 *Fpr3-GFP* and 1224 control-GFP cells, in 12, 8 and 27 experiments, respectively. Scale bars, 10 μm.

Our results described thus far indicate that ectopic overexpression of a given *V1R* in VSNs via HSV-1 mediated gene transfer results in a greatly increased number of VSNs that respond to specific ligands for this receptor (Fig 3). On the basis of these results, we reasoned that overexpression of other types of vomeronasal receptors including *V2R*s and *Fpr*s should also lead to increased responsiveness to specific ligands recognized by these receptors. To determine whether this is the case, we cloned the receptor *V2r1b* (*Vmn2r24*) into the HSV-1 amplicon and monitored Ca^2+^ responses to the class I MHC binding peptide SYFPEITHI. Previous studies in VNO tissue slices from *V2r1b*-IRES-*tauGFP* gene-targeted mice revealed that more than half of VSNs expressing this receptor are normally activated by SYFPEITHI [10,11]. Remarkably, in our *V2r1b-GFP* virus expression system, we observed a clear 6-fold increase in the number of cells responding to SYFPEITHI (10^−11^ M) stimulation versus receptor-free GFP controls (p < 0.005) (Fig 4A and 4B). By contrast, other related ligands such as the mitochondria-derived formylated peptide f-MFFINTLTL (ND1) and ligands present in the HMW fraction of urine did not produce enhanced responsiveness (p = 0.37–0.65) (Fig 4B). These results strongly argue that our virus-based expression approach can not only be used for *V1R*s but also for *V2R*s.

Both ND1 and W-peptide are known to function as Fpr agonists [13]. *Fprs* are expressed in the VNO by a small subset of VSNs which do not seem to overlap with *V1R*- or *V2R*-expressing VSNs [20]. To assess whether virus-mediated overexpression could serve to functionally express *Fprs*, we infected VSNs with a HSV-1 amplicon encoding *Fpr3*. This receptor is specifically activated by W-peptide but not by ND1 [13]. Using Ca^2+^ imaging we found that W-peptide activated a greatly increased number of *Fpr3-GFP* infected cells, by ~20 fold (p < 0.001) compared to GFP controls (Fig 4D and 4E), whereas ND1 and HMW fraction failed to induce higher levels of activity (p = 0.83–0.63) (Fig 4E). Hence, HSV-1-mediated gene expression is also applicable to *Fpr3*.

Previous studies have shown that electrical and Ca^2+^ responses in the VNO evoked by SYFPEITHI and some formylated peptides require the participation of the G-protein subunit Gαo [22]. To gain insight into the signaling properties of the overexpressed *V2r1b* receptor and to obtain additional controls about the specificity of our expression system, we used VSNs obtained from the Gnao1/OMP-Cre conditional Gαo knockout mouse line [22–24]. When such VSNs were infected with *V2r1b-GFP* virus, Ca^2+^responses to SYFPEITHI were drastically reduced (2 out of 193 cells; ~1%) (Fig 4C), now reaching similar values (p = 0.96) as those evoked by HMW and ND1 (2 and 3 cells). This result indicates that the presence of Gαo is required for the generation of enhanced responsiveness to SYFPEITHI in VSNs after virus-mediated *V2r1b* expression. Importantly, Gαo-deficient VSNs showed normal Ca^2+^ transients to high K^+^ solution, indicating intact viability and excitability (Fig 4C).

## Discussion

We have developed a virus-based gene delivery procedure to construct an expression system for the investigation of receptor-ligand interactions in the VNO. By choosing a selected member from each of the three major vomeronasal receptor families–*V1rj2*, *V2r1b* and *Fpr3* –we demonstrate here that virus-induced overexpression in native VNO cells is a useful approach to characterize and identify receptor-ligand pairs underlying molecular sensing in the VNO. In each case, receptor overexpression resulted in a markedly increased number of infected cells responding to specific ligands. These ligands have been linked to these receptors previously, either through heterologous expression [13,14] or through the use of genetically modified mouse lines [7,10,11]. Together with the direct expression system developed here, these results provide a firm foundation for understanding the detection capabilities of specific vomeronasal receptors.

Neither viral infection nor GFP alone were sufficient to elicit this gain in responsivity and even closely related ligands were not recognized by the overexpressed receptor, indicating a high specificity of our system. Because the infected cells expressed GFP, it was possible to identify individual positive neurons, allowing Ca^2+^ responses to be tested in single cells (Figs 3 and 4). The ligand response in virally-transduced cells followed the pattern of transient intracellular Ca^2+^ increases shown in untreated cells (Fig 1A and [7,10,22,25,36,37]), indicating that the endogenous second messenger system expressed by these neurons was most likely responsible for generating the response in the infected cells. This view is further strengthened by the observation that infection of VSNs lacking Gαo—a key transduction molecule in basal VSNs [22]–with viruses encoding the *V2r1b* receptor failed to show an increase in the responses to SYFPEITHI peptide (Fig 4C).

We observed a 6- to 20-fold gain in responsivity to specific ligands after infection, but the overall number of activated cells was relatively low (about 3–5%), even though as many as 76% of infected cells were neurons (Fig 2E). The reasons for this are not known, but the most parsimonious explanation would be limited access to the correct endogenous second messenger and trafficking systems for membrane expression. Only half of the neurons express a given G-protein, either Gαi2 or Gαo, and correct localization to the plasma membrane may require extra components specific for particular receptors or receptor families. This could be the case for at least certain V2Rs that seem to require calreticulin4 and/or other factors to display fully functional responsive features [11,12]. It is also conceivable that some vomeronasal receptors may form heterodimers or heteromultimers [10,38]. Thus, the possible interaction of the overexpressed receptor with an endogenous receptor may have an impact on the activation profile of infected cells. Further experiments overexpressing different receptor types simultaneously in the same cells will be needed in the future to address these questions.

Matching the immense repertoire of vomeronasal receptors to the even greater collection of putative stimuli (the vomeronasal ligandome) and systematic analysis of ligand-receptor interactions are fundamental steps towards understanding how chemical information is encoded by the olfactory systems. Future identification of ligand-receptor pairs will define chemical coding mechanisms during social communication, the neural circuits that are activated by specific receptors and their ligands, and their precise roles in the control of social behaviors.

## Supporting Information

S1 File Supporting data. Supporting Information Excel workbook file containing the raw data from Figs 2D, 2E, 3C–3E, 4B, 4C and 4E.
(XLSX)
